# The systemic-immune-inflammation index predicts the recurrence of atrial fibrillation after cryomaze concomitant with mitral valve surgery

**DOI:** 10.1186/s12872-022-02494-z

**Published:** 2022-02-13

**Authors:** Yu Luo, Jian Zhang, Tao Liu, Zongtao Yin, Yan Jin, Jinsong Han, Zhipeng Guo, Huishan Wang

**Affiliations:** 1 Department of Cardiovascular Surgery, General Hospital of Northern Theater Command, No.83, Wenhua Road, Shenhe District, Shenyang, 110016 Liaoning China; 2 ICU of Surgery, Wuhan Asia Heart Hospital, Jianghan District, Wuhan, 430040 Hubei China; 3 Postgraduate Training Base of Northern Theater Command General Hospital, China Medical University, No.83, Wenhua Road, Shenhe District, Shenyang, 110016 Liaoning China

**Keywords:** Systemic-immune-inflammation index, Recurrence of atrial fibrillation, CryoMaze, Systemic immune-inflammation index

## Abstract

**Background and aims:**

Inflammation plays a key role in the initiation and progression of atrial fibrillation (AF). The systemic inflammation indexes are easily evaluated and predict AF development. However, it’s role in prediction of recurrence of AF is unknown. We aim to explore the association between the systemic inflammation indexes and recurrence of AF in patients underwent cryoablation (CryoMaze) concomitant with mitral valve surgery.

**Methods:**

We examined systemic inflammation indexes during perioperative period in 122 patients between 2015 and 2018. Systemic inflammation indexes were developed by systemic immune-inflammation index (SII), neutrophil to lymphocyte ratio (NLR), platelet to lymphocyte ratio (PLR), and lymphocytes to monocytes ratio. Univariate and multivariate analyses were performed to examine the association of each markers with recurrence of AF.

**Results:**

Of the 122 patients included in this study, 22 patients (18%) experienced AF recurrence after CryoMaze concomitant with mitral valve surgery. There is no significant difference between each systemic inflammation indexes before surgery and recurrence of AF. In univariate analysis, MLR after surgery 3 days, PLR, MPLR, NLR, SII after surgery 7 days were able to predict recurrence of AF. In multivariate analyses, SII ≥ 1696 independently predicted recurrence (OR, 3.719; 95% CI, 1.417–9.760). Interestingly, baseline SII showed no significant in prediction of recurrence. It was sharply elevated after surgery and dropped slowly. In patients of recurrence, SII after 7 days of surgery increased again.

**Conclusions:**

The raised SII again was associated with an increased risk of the postoperative recurrence of AF and independently predicted the late recurrence of AF after CryoMaze concomitant with mitral valve surgery.

**Supplementary Information:**

The online version contains supplementary material available at 10.1186/s12872-022-02494-z.

## Introduction

Atrial fibrillation (AF) is a common arrhythmia associated with mitral valve disease. Nearly half of patients undergoing mitral valve surgery frequently present with AF, which is associated with poor prognosis [1]. The Cox-Maze IV operation is widely used in the surgical treatment of AF [2]. However, the efficiency was various [3–5]. Our recent study indicated that the efficiency of cryoablation (CryoMaze) concomitant with mitral valve surgery was achieved in 85% (95% CI, 0.76–0.91) [5].

The pathophysiological mechanisms of AF are not well known [6]. Inflammation and substrate alterations (such as fibrosis) are complex and critical for the understanding of AF [7–10]. The mechanism of recurrences may because of incomplete isolation, acute inflammatory changes, recovery of conduction and modification of autonomic nervous system [11]. The systemic inflammation index is a systemic inflammation based on routine blood tests [12]. Neutrophil to lymphocyte ratio (NLR), platelet to lymphocyte ratio and lymphocyte to monocyte ratio (LMR) are reported to be associated with systemic inflammation status and AF progression [13]. Systemic immune-inflammation index (SII) is a novel marker that brings together these three inflammatory peripheral cell counts and predict functionally significant coronary artery stenosis [14–16]. In this study, we investigated the association of perioperative systemic inflammation index, and changes during treatment, with outcomes in AF patients treated with CryoMaze concomitant with mitral valve surgery.

## Methods

### Patients’ selection

We retrospectively studied consecutive 150 long-persistent or persistent AF patients who required to undergoing the cryoMaze procedures combined with valve-surgery at the department of cardiovascular surgery, General Hospital of Northern Theatre Command, from 2015 to 2018. Exclusion criteria were (1) emergency surgery; (2) combined with CABG or any other heart procedures; (3) previous cardiovascular surgery; (4) primary pulmonary hypertension; (5) EF < 0.40, and (6) history of cerebral hemorrhage or brain stoke within 3 months. Of these, 122 (81.3%) had baseline complete blood counts necessary for the inflammatory indexes and all clinical data available and were considered fully evaluable for this post hoc analysis.

This study was approved by the General Hospital of Northern Theatre Command’s Ethics committee. All procedures were performed by the Declaration of Helsinki and its later amendments or comparable ethical standards.

### Surgical procedure, ablation procedure, post-operative management, and follow-up

Surgical procedure, ablation procedure, post-operative management and follow-up have been described before [5]. Briefly, after cardioplegic arrest and aortic cross-clamping, the left lesion sets and concomitant operations were performed. The lesion sets of the CryoMaze were created using cryothermia based on Nitrous Oxide (Atricure CRYO2 Cryosurgical probe, − 60 °C, 2 min). The detail of lesion sets, and ablation procedure could be found before [5].

Heart rhythm was monitored continuously throughout the hospital stay, and temporary epicardial wires were used for ventricular pacing as needed in all the patients after the surgery. Amiodarone was given intravenously from 20 to 40 mg/h, followed by oral amiodarone at 200 mg twice a day and then 200 mg/day until 3 months after discharge. According to the 24-h Holter results at 3 months, amiodarone was withdrawn for patients restore to sinus rhythm. Amiodarone was continually administered in AF patients. Electrical cardioversion was applied in patients when oral amiodarone failed to maintain sinus rhythm. After discharge, patients were followed up at outpatient clinic at 1, 3, 6, and 12 months. Heart rhythm was verified with 24-h Holter monitoring and echocardiography were evaluated by 2-dimensional echocardiographic analysis and Doppler color flow imaging (Philips iE33 ultrasound machine; Philips Healthcare, Andover, Mass) at each visit [5]. The definition of atrial tachyarrhythmia recurrence was any documented AF, atrial flutter, or atrial tachyarrhythmia lasting ≥ 30 s after 3 months blanking period [17].

### Post hoc analysis variable definitions

For this post hoc analysis, the inflammatory indexes were determined based on values of monocytes (M), neutrophils (N), lymphocytes (L), and/or platelets (P) in patients received surgical ablation at indicated data: SII defined as P × N/L, NLR defined as N/L, platelet-lymphocyte ratio (PLR) defined as P/L, lymphocyte to monocyte ratio (LMR) defined as L/M, monocytes-NLR (MNLR) defined as M × N/L, monocytes-PLR (MPLR) defined as M × P/L [14, 18, 19].

### Statistical analysis

The IBM SPSS.24.0 software was used for all dates analyzed. Continuous variables were presented as Mean ± SD or medians (range). And the categorical variables were described as frequencies and percentages. To compare the differences between two groups, the independent student’s *t* text and Mann–Whitney U text were used. Chi-squared test or Fisher’s exact test were used for categorical variables. Risk factors that *p* < 0.05 were included in the Univariate logistic regression analysis. Receiver operating characteristic curves (ROC) were generated to provide data on the predictive ability of the systemic inflammation indexes to detect recurrence of AF. The area under the curve (AUC) was used to quantify the ROC curve. Youden’s index (J = Sensitivity + Specificity − 1) was used to determine the most appropriate cut-off value. *p* < 0.05 was considered significant in all comparisons.

## Results

### Study population

A total of 122 patients who underwent CryoMaze concomitant with mitral valve surgery were included in this study. Recurrence occurred in 22 patients (18.0%). Table 1 summarizes the characteristics of the cohort based on recurrence or not. Preoperative left atrial diameter (LAD) (*p* = 0.196) and left atrial volume indexed (LAVI) (*p* = 0.229) are elevated in patients have AF recurrence, but there was no statistical significance. All of patients were diagnosed with NYHA III (N = 100) or IV (N = 22) and there was no significance between two groups.

**Table 1:** Baseline and clinical characteristics (N = 122)

	Rhythm after surgery (12 m)		*P*
	Recurrence versus	Non-recurrence	
Age	59.640 ± 6.403	59.690 ± 8.781	0.978
Gender (%) (Male versus female)	11 (50.0) versus 9 (50.0)	32 (32.0) versus 68 (68.0)	0.110
Degenerative disease (%)	9 (40.9)	47 (47.0)	0.604
Hypertension (%)	4 (18.2)	13 (13.0)	0.768
Coronary arteries disease (%)	6 (27.3)	28 (28.0)	0.945
Diabetes (%)	2 (9.1)	5 (5.0)	0.810
Kidneys disease (%)	0 (0)	3 (3.0)	1.000
NYHA III (%)	15 (68.2%)	70 (70.0%)	0.867
LAD (mm)	54.500 ± 9.787	51.600 ± 8.661	0.168
LAVI (ml/m^2)^	47.320 ± 8.231	45.080 ± 7.782	0.229
LVEDD (mm)	50.590 ± 6.345	47.750 ± 6.363	0.060
LVEDV (ml)	153.140 ± 147.128	111.000 ± 36.166	0.196
LVEF (%)	54.000 ± 5.800	56.000 ± 4.200	0.192
ACC (min)	83.180 ± 25.299	87.440 ± 31.255	0.552
CPB (min)	143.820 ± 35.391	146.540 ± 42.790	0.782

*LAD* left atrium diameter, *LAVI* left atrial volume indexed, *LVEDD* left ventricular end diastolic diameter, *LVEDV* left ventricular end diastolic volume, *LVEF* left ventricular ejection fraction, *ACC* aortic clip, *CPB* cardiopulmonary bypass

### Systemic inflammation index and recurrence

**Fig. 1:**
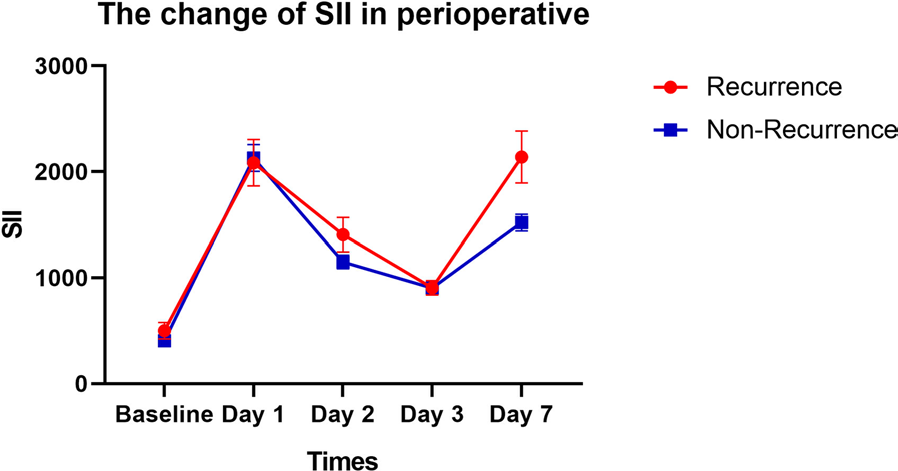
The tendency of SII before and after surgery at indicated days

The systemic inflammation index before surgery was no related with recurrence (Additional file 1: Table S1). Furthermore, the similar results were found in the early after the surgery (Additional file 1: Tables S2, S3 and S4). Interestingly, NLR, PLR, MPLR and SII levels after 7 days of surgery were significantly higher in recurrence group (*p* = 0.033; *p* = 0.050; *p* = 0.042; *p* = 0.024) (Table 2). Furthermore, we found that in recurrence patients SII dropped along with no-recurrence patients after 1 day of surgery but elevated at 7 days after the surgery (Fig. 1).

**Table 2:** Inflammatory markers of day 7 in post-operative

	Rhythm after surgery (12 m)		*P*
	Recurrence versus	Non-recurrence	
NLR-7	6.869 ± 2.615	5.667 ± 2.310	**0.033**
MLR-7	0.619 ± 0.257	0.568 ± 0.197	0.301
PLR-7	241.901 ± 99.112	201.624 ± 83.667	**0.050**
MPLR-7	185.928 ± 100.588	150.150 ± 66.969	**0.042**
MNLR-7	5.456 ± 2.962	4.452 ± 2.402	0.092
SII-7	2137.871 ± 1150.615	1520.137 ± 786.779	**0.024**
ALB-7	33.031 ± 2.352	33.960 ± 3.647	0.256

*NLR-7* the NLR of post-day 7, *MLR-7* the MLR of post-day 7, *PLR-7* the PLR of post-day 7, *MPLR-7* the MPLR of post-day 7, *MNLR-7* the MNLR of post-day 7, *SII-7* the SII of post-day 7, *ALB-7* the albumin of post-day 7

Bold values indicated *p* < 0.05

**Fig. 2:**
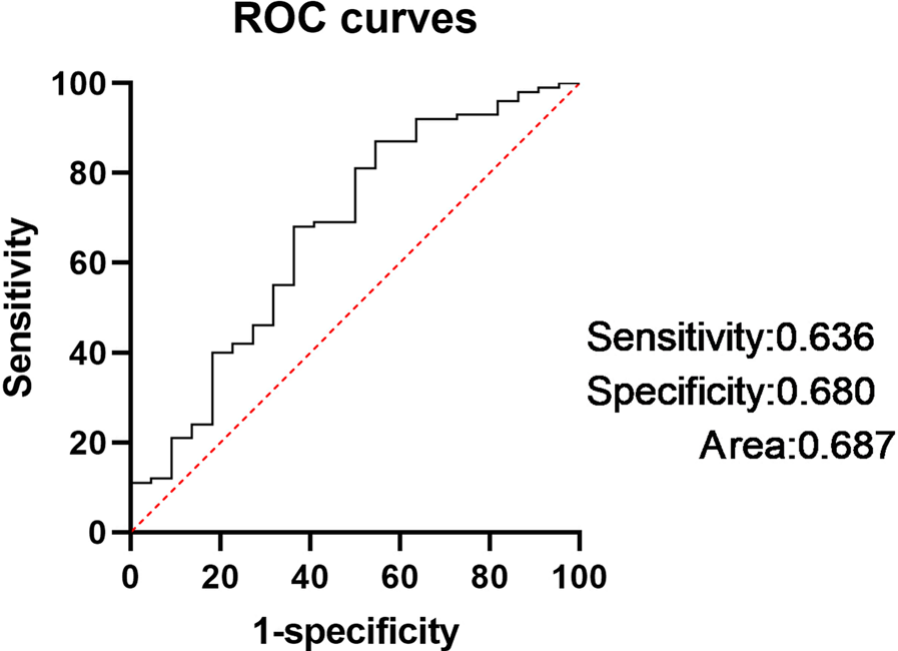
ROC curve analysis to determine the predictive value of SII for AF recurrence. *AF* atrial fibrillation, *AUC* area under the curve, *ROC* receiver operating characteristic

We used SII at 7 days after surgery data for receiver operating characteristic (ROC) analysis. The area under the ROC curve was 0.680, and the 95% confidence interval was 0.566–0.808(Fig. 2). A cut-off points of 1696 was related to recurrence with a sensitivity of 63.6% and a specificity of 68.0%.

The distribution of characteristics in cohort and SII levels after 7 days of surgery is listed in Additional file 1: Table S5. However, there was no correlation between high SII levels after 7 days of surgery and other variables (Additional file 1: Table S5).

### Univariate and multivariate analysis of recurrence

On multivariable logistic regression analysis, high SII after 7 days of surgery (OR, 3.719 (1.417–9.760), *p* = 0.008) were significant and independent risk factors for recurrence (Table 3).

**Table 3:** Univariable and multivariable logistic regression of SII-7

	Univariable			Multivariable		
	OR	*p*	CI	OR	*p*	CI
High MLR-3	2.855	**0.032**	1.096–7.442			
High PLR-7	3.127	0.053	0.986–9.919			
HighMPLR-7	3.380	**0.017**	1.247–9.163			
High NLR-7	3.810	**0.008**	1.422–10.208			
High SII-7	3.719	**0.008**	1.417–9.760	3.719	**0.008**	1.417–9.760

*High MLR-3* the MLR of postoperative day 3 ≥ 0.63961, *High PLR-7* the PLR of postoperative day 7 ≥ 174.2083, *High MPLR-7* the MPLR of postoperative day7 ≥ 189.9917, *High NLR-7* the NLR of postoperative day 7 ≥ 5.913194, *High SII-7* the SII of postoperative day7 ≥ 1696.005051, *OR* odds ratio, *CI* confidence interval; The variables of Multivariable are High MLR-3, HighMPLR-7, High NLR-7, High SII-7

Bold values indicated *p* < 0.05

## Discussion

In our retrospective study, we focused more on the relationship between the systemic inflammation index and development of AF recurrence after CryoMaze concomitant with mitral valve surgery. We found that SII was increased immediately after the surgery and dropped within several days. Interestingly, in recurrence patients, SII levels elevated at 7 days after surgery again. Additionally, we had demonstrated that the SII are independent prognostic factor of AF recurrence in patients who had undergone CryoMaze concomitant with mitral valve surgery.

The precise mechanisms of AF recurrence have not been fully elucidated. it is well established that inflammation is independently associated with the development and recurrent of AF [20]. Several studies indicated markers of inflammation predicted recurrence of AF [21, 22]. The atrial tissue changes such as fibrosis, leukocyte infiltrates and oxidative damage may contribute to electrical and structural remodeling, which may promote recurrence of AF [23]. Some inflammatory biomarkers, including CRP, IL-6, IL-2, matrix metalloproteinase-2 and tumor necrosis factor-α, were found to be significantly associated with recurrent AF [21, 24, 25]. Moreover, anti-inflammatory therapy may reduce the recurrence AF [26].

The SII can better reflect the immune and inflammatory state of the body compared to the use of any one of these markers in isolation [27]. It has been widely studied in different cancers [12, 27]. What is more, levels of neutrophils and lymphocytes are related with initiation and progression of AF [28–30]. Inflammatory mediators, released by neutrophils, such as interleukin-8, matrix metalloproteinase-9, and vascular endothelial growth factor promoted AF. Recently, platelets was reported the profibrotic actions through Transforming Growth Factor-β1 dependent manners [31]. SII not only contained the ratio of neutrophil and lymphocyte ratio, but contained the levels of platelets, which may reflect atrial fibrosis to some extent. Therefore, SII is superior to other systemic inflammation index in prognostic assessment of AF ablation in our cohort.

AF is the most common cardiac arrhythmia worldwide. In patients underwent valve surgery, the prevalence of AF is approximately 30–50% [5]. The cut-and-sew Maze procedure is considered the gold standard for the surgical treatment of AF [32]. Because of its complexity and perceived risks of bleeding, various energy sources such as radiofrequency and cryoablation to simplify surgical AF ablation. Previously, our group reported the addition of the Maze performed by cryoablation (CryoMaze) was non-inferior to CSM for efficacy and safety for patients with persistent or long-standing persistent AF undergoing mitral valve surgeries [5]. The efficacy reported by us is similar with that reported by other groups [33]. So, the recurrence of AF is not blame for our procedure. Many evidences indicated acute inflammatory changes is essential for recurrence of AF [11]. Our results clearly showed SII was elevated at 1 day after the surgery, then it dropped significantly. However, in patients who would fail to ablation, SII was elevated again. Our finding supported inflammatory changes promoted AF recurrence. On the other hand, targeting local inflammation is a therapeutic option for preventing recurrences of AF. Although, inflammation is not the only factor responsible for triggering recurrences of AF [34]. Anti-inflammatory such as colchicine were consistent and promising for AF recurrence [34]. All this evidence indicated targeting inflammatory could be one method for AF recurrence in patients treated with CryoMaze concomitant with mitral valve surgery.

This was a single center retrospective analysis of a small patient cohort. In addition, the relatively small sample size in this study limited the statistical capacity. Further prospective studies would be need to confirmation of these findings with large sample size.

In conclusion, SII as an inflammatory marker is associated with an independent increased risk of AF recurrence among patients underwent CryoMaze concomitant with mitral valve surgery. Future research is needed to elucidate the specific mechanism of inflammatory biomarkers in the recurrence of AF.

## Supplementary Information

**Additional file 1:** Inflammatory markers in per-operative period.

## Data Availability

The essential data are available from the corresponding author on reasonable request.

## Abbreviations

AF: Atrial fibrillation
CryoMaze: Cryoablation
LAD: Left atrial diameter
LMR: Lymphocytes to monocytes ratio
LVEDD: Left ventricular end diastolic diameter
NLR: Neutrophil to lymphocyte ratio
PLR: Platelet to lymphocyte ratio
ROC: Receiver operating characteristic
SII: Systemic immune-inflammation index
