# Supplementary material for: The systemic-immune-inflammation index predicts the recurrence of atrial fibrillation after cryomaze concomitant with mitral valve surgery
Source: BMC Cardiovasc Disord. 2022 Feb 13;22:45. doi: 10.1186/s12872-022-02494-z (PMC8842953; doi:10.1186/s12872-022-02494-z)
Supplement: Supplementary file 1 — Additional file 1: Inflammatory markers in per-operative period. [file 12872_2022_2494_MOESM1_ESM.doc]

The Systemic-immune-inflammation Index predicts the recurrence of atrial fibrillation after cryomaze concomitant with mitral valve surgery

Yu Luo, ^1,2 † MM^, Jian Zhang, ^1, † MD^, Tao Liu, ^1, † MM^, Zongtao Yin ^1, MD^, Yan Jin ^1, MD^, Jinsong Han, ^1, MD^, Zhipeng Guo,^1, 3, MM^, Huishan Wang ^1 * MD, PhD^

Running Title: Systemic-immune-inflammation Index and recurrence of atrial fibrillation

Affiliations:

1 Department of Cardiovascular Surgery, General Hospital of Northern Theater Command, No.83, Wenhua Road, Shenhe District, Shenyang, Liaoning,110016 China.

2. ICU of Surgery, Wuhan Asia Heart Hospital, Jianghan District, Wuhan, Hubei, 430040 China.

3. Postgraduate Training Base of Northern Theater Command General Hospital，China Medical University, No.83, Wenhua Road, Shenhe District, Shenyang, Liaoning,110016 China.

Corresponding Authors:

* Huishan Wang and Jinsong Han: Department of Cardiovascular Surgery, General Hospital of Northern Theater Command, No.83, Wenhua Road, Shenhe District, Shenyang, Liaoning,110016 China. Phone: +862428897382; Fax: +862423912376; Email: huishanw@126.com and hanjs0216@sina.com;

†, These authors contributed equally to this work.

Keywords: Systemic-immune-inflammation index; Recurrence of atrial fibrillation; CryoMaze; Systemic immune-inflammation index

Table S1: Inflammatory markers in per-operative period

|  | Rhythm after surgery（12m） | | *P* |
| --- | --- | --- | --- |
|  | Recurrence *VS* | Non-recurrence |  |
| NLR | 2.770±2.255 | 2.140±1.380 | 0.091 |
| MLR | 0.288±0.184 | 0.258±0.144 | 0.407 |
| PLR | 117.739±75.517 | 106.389±59.745 | 0.444 |
| MPLR | 54.051±38.894 | 48.512±29.078 | 0.450 |
| MNLR | 1.265±0.875 | 0.980±0.664 | 0.090 |
| SII | 501.866±363.009 | 405.696±289.371 | 0.181 |
| ALB | 41.105±3.040 | 39.582±3.734 | 0.077 |

NLR：Neutrophil/Lymphocyte Ratio；MLR：Monocyte/ Lymphocyte Ratio；PLR：Platelet/ Lymphocyte Ratio；MPNLR：Monocyte * Platelet / Lymphocyte Ratio；MNLR：Monocyte * Neutrophil / Lymphocyte Ratio; SII：the systemic immune-inflammatory index，Neutrophil* Platelet / Lymphocyte Ratio；ALB：Albumin

Table S2 : Inflammatory markers of day 1 in post-operative

|  | Rhythm after surgery（12m） | | *P* |
| --- | --- | --- | --- |
|  | Recurrence *VS* | Non-recurrence |  |
| NLR | 19.644±10.294 | 18.922±9.389 | 0.749 |
| MLR | 1.155±0.934 | 0.999±0.484 | 0.262 |
| PLR | 173.891±80.265 | 178.181±84.491 | 0.828 |
| MPLR | 122.443±85.655 | 114.697±69.511 | 0.651 |
| MNLR | 14.113±11.635 | 12.307±8.854 | 0.416 |
| SII | 2084.729±1022.157 | 2129.570±1263.373 | 0.877 |
| ALB | 27.773±3.581 | 27.846±4.239 | 0.940 |

NLR：Neutrophil/Lymphocyte Ratio；MLR：Monocyte/ Lymphocyte Ratio；PLR：Platelet/ Lymphocyte Ratio；MPNLR：Monocyte * Platelet / Lymphocyte Ratio；MNLR：Monocyte * Neutrophil / Lymphocyte Ratio; SII：the systemic immune-inflammatory index，Neutrophil* Platelet / Lymphocyte Ratio；ALB：Albumin

Table S3: Inflammatory markers of day 2 in post-operative

|  | Rhythm after surgery（12m） | | *P* |
| --- | --- | --- | --- |
|  | Recurrence *VS* | Non-recurrence |  |
| NLR | 13.129±5.913 | 11.660±6.861 | 0.354 |
| MLR | 0.906±0.291 | 0.791±0.308 | 0.113 |
| PLR | 117.553±40.789 | 109.063±48.913 | 0.450 |
| MPLR | 96.578±39.842 | 79.961±38.058 | 0.068 |
| MNLR | 10.958±6.085 | 8.902±5.757 | 0.136 |
| SII | 1405.924±759.953 | 1149.346±639.350 | 0.102 |
| ALB | 29.968±3.314 | 30.516±2.873 | 0.432 |

NLR：Neutrophil/Lymphocyte Ratio；MLR：Monocyte/ Lymphocyte Ratio；PLR：Platelet/ Lymphocyte Ratio；MPNLR：Monocyte * Platelet / Lymphocyte Ratio；MNLR：Monocyte * Neutrophil / Lymphocyte Ratio; SII：the systemic immune-inflammatory index，Neutrophil* Platelet / Lymphocyte Ratio；ALB：Albumin

TableS4: Inflammatory markers of day 3 in post-operative

|  | Rhythm after surgery（12m） | | *P* |
| --- | --- | --- | --- |
|  | Recurrence *VS* | Non-recurrence |  |
| NLR | 8.149±3.916 | 6.917±3.374 | 0.135 |
| MLR | 0.701±0.207 | 0.600±0.202 | 0.038 |
| PLR | 128.857±47.620 | 120.474±62.179 | 0.553 |
| MPLR | 94.654±43.293 | 80.999±44.172 | 0.190 |
| MNLR | 6.115±3.343 | 4.747±2.850 | 0.051 |
| SII | 906.746±314.045 | 903.063±521.346 | 0.975 |
| ALB | 32.368±3.211 | 36.295±38.104 | 0.631 |

NLR：Neutrophil/Lymphocyte Ratio；MLR：Monocyte/ Lymphocyte Ratio；PLR：Platelet/ Lymphocyte Ratio；MPNLR：Monocyte * Platelet / Lymphocyte Ratio；MNLR：Monocyte * Neutrophil / Lymphocyte Ratio; SII：the systemic immune-inflammatory index，Neutrophil* Platelet / Lymphocyte Ratio；ALB：Albumin

Table S5: The Baseline and clinical characteristics in different level of SII

|  | High-SII  *VS*  （N=46） | Low-SII  （N=76） | *P* |
| --- | --- | --- | --- |
| Age | 59.800±8.881 | 59.610±8.119 | 0.899 |
| Gender  (male %) | 17(36.9) | 26（34.2) | 0.758 |
| Degenerative Disease（%） | 27（58.6） | 39（51.3） | 0.428 |
| Hypertension（%） | 7（9.2） | 10（13.1） | 0.750 |
| Coronary Arteries Disease（%） | 13（28.2） | 21（27.6） | 0.940 |
| Diabetes（%） | 0（0） | 7（9.2） | 0.086 |
| Kidneys Disease（%） | 1（2.1） | 2（2.6） | 1.000 |
| NYNA III (%) | 33 (71.3) | 52 (68.4) | 0.699 |
| LAD（mm） | 52.910±9.874 | 51.640±8.291 | 0.448 |
| LAVI (mL/m2) | 46.350± 8.805 | 44.960±7.271 | 0.348 |
| LVEDD（mm） | 48.480±6.124 | 48.130±6.642 | 0.774 |
| LVEDV（ml） | 114.760±37.483 | 120.920±86.525 | 0.646 |
| LVEF(%) | 55.000±4.800 | 55.000±4.500 | 0.688 |
| ACC（min） | 89.170±33.818 | 85.160±27.940 | 0.479 |
| CPB (min) | 149.910±48.292 | 143.710±36.816 | 0.425 |

LAD：Left atrium diameter ；LVEDD：Left ventricular end diastolic diameter ;LVEDV: Left ventricular end diastolic volume；LVEF: Left ventricular ejection fraction；ACC: Aortic clip；CPB: Cardiopulmonary bypass

| Markers | Sensitivity | Specificity | Area | *P* value | 95%CI | Cut-off |
| --- | --- | --- | --- | --- | --- | --- |
| MLR-3 | 0.636 | 0.620 | 0.648 | 0.030 | 0.230-0.772 | 0.639 |
| NLR-7 | 0.682 | 0.640 | 0.643 | 0.036 | 0.513-0.773 | 5.913 |
| PLR-7 | 0.818 | 0.410 | 0.620 | 0.079 | 0.492-0.748 | 174.208 |
| MPLR-7 | 0.409 | 0.830 | 0.596 | 0.158 | 0.462-0.730 | 189.991 |
| SII-7 | 0.636 | 0.680 | 0.687 | 0.013 | 0.566-0.808 | 1696.005 |

Table S6: The ROC curves information of different markers

Table S7: The diagnosis of multi-collinearity in the markers

| Markers | VIF |
| --- | --- |
| Constant |  |
| NLR-7 | 2.575 |
| PLR-7 | 2.257 |
| SII-7 | 4.971 |
| MPLR-7 | 2.412 |
